# Relationship between carotid plaque surface morphology and perfusion: a 3D DCE-MRI study

**DOI:** 10.1007/s10334-017-0621-4

**Published:** 2017-04-28

**Authors:** Jianmin Yuan, Gregory Makris, Andrew Patterson, Ammara Usman, Tilak Das, Andrew Priest, Zhongzhao Teng, Sarah Hilborne, Dario Prudencio, Jonathan Gillard, Martin Graves

**Affiliations:** 1 Department of Radiology, University of Cambridge, Cambridge, UK; 2 Department of Radiology, Cambridge University Hospitals NHS Foundation Trust, Cambridge, UK

**Keywords:** Dynamic contrast enhanced MRI (DCE-MRI), Contrast enhanced MR angiography (CE-MRA), Carotid atherosclerotic plaque, Plaque ulceration, Neovascularization

## Abstract

**Objective:**

This study aims to explore the relationship between plaque surface morphology and neovascularization using a high temporal and spatial resolution 4D contrast-enhanced MRI/MRA sequence.

**Materials and methods:**

Twenty one patients with either recent symptoms or a carotid artery stenosis ≥40% were recruited in this study. Plaque surface morphology and luminal stenosis were determined from the arterial phase MRA images. Carotid neovascularization was evaluated by a previously validated pharmacokinetic (PK) modeling approach. *K* ^trans^ (transfer constant) and *v*_p_ (partial plasma volume) were calculated in both the adventitia and plaque.

**Results:**

Image acquisition and analysis was successfully performed in 28 arteries. Mean luminal stenosis was 44% (range 11–82%). Both adventitial and plaque *K* ^trans^ in ulcerated/irregular plaques were significantly higher than smooth plaques (0.079 ± 0.018 vs. 0.064 ± 0.011 min^−1^, *p* = 0.02; 0.065 ± 0.013 vs. 0.055 ± 0.010 min^−1^, *p* = 0.03, respectively). Positive correlations between adventitial *K* ^trans^ and *v*_p_ against stenosis were observed (*r* = 0.44, *p* = 0.02; *r* = 0.55, *p* = 0.01, respectively).

**Conclusion:**

This study demonstrates the feasibility of using a single sequence to acquire both high resolution 4D CE-MRA and DCE-MRI to evaluate both plaque surface morphology and function. The results demonstrate significant relationships between lumen surface morphology and neovascularization.

## Introduction

Stroke is one of the leading causes of death and disability worldwide [1] with ischemic strokes accounting for 90% of the total reported cases [2]. Twenty percent of ischemic strokes are attributed to carotid atherosclerosis [3]. Carotid luminal stenosis measured by ultrasound plays an important role in screening high risk patients and is the standard criteria for surgical referral [4]. Whilst only patients with severe stenosis (≥70%) have shown a benefit from carotid endarterectomy (CEA), patients with moderate stenosis (<70%) have only shown marginal beneficial effects from surgical treatment [5, 6]. There is an increasing recognition that other factors, such as plaque surface morphology, may have higher predictive value in moderately stenosed plaques [7].

The appearance on X-ray angiography of plaque surface irregularity, or ulceration, has been correlated with increased risk of ischemic stroke [8]. This correlation applies for both moderate and severe stenosis [8]. Carotid ulceration is found to be more common in patients with downstream micro emboli [9]. Ulceration has also been associated with high risk factors including plaque rupture, intraplaque hemorrhage and a large lipid-rich necrotic core (LRNC) [10], indicating this is a sensitive marker of plaque instability [10] and risk of stroke [8, 11, 12]. Noninvasive imaging modalities offer high accuracy for ulcer detection as well as stenosis measurement [13–15]. Among different imaging modalities, contrast-enhanced MR angiography provides high resolution imaging allowing visualization of plaque ulceration [13, 16, 17].

In this study we have applied a 4D sequence [18] which acquires data suitable for contrast enhanced (CE) MRA and dynamic contrast enhanced (DCE)-MRI processing. DCE-MRI has been demonstrated as a non-invasive method to quantify the extent of neovasculature within carotid plaques with high accuracy and reproducibility [19, 20]. Previous studies using DCE-MRI have shown that plaque neovascularization is closely associated with high risk features, such as inflammation [21], and is related to ischemic events [22]. In this study, we investigated the correlation between plaque surface morphology and neovascularization.

## Materials and methods

### Experimental methods

The study protocol was reviewed and approved by the local ethics committee and written informed consent was obtained. Twenty one patients who had a carotid stenosis of at least 40% on Duplex ultrasound using NASCET criteria [23] were recruited in total. Ten were symptomatic (nine males, mean age 72 years, range 59–86 years) with a recent history (less than 6 months) of transient ischemic attack (TIA), and 11 were asymptomatic (seven males, mean age 78 years, range 68–87 years).

### MRI protocol

Imaging was performed on a 3T system (MR750, GE Healthcare, Waukesha, WI, USA), using a 4 channel phased-array neck coil (PACC, MachNet, Roden, The Netherlands). The time-resolved imaging of contrast kinetics (TRICKS) method [18] was performed to obtain 4D CE-MRI with the acquisition resolution of 0.6 × 0.6 × 1.4 mm^3^ and the images were interpolated into 0.3 × 0.3 × 0.7 mm^3^ for the DCE analysis. A coronal imaging slab was centered on the carotid bifurcation of the higher stenosis degree side. The coronal acquisition plane was chosen to minimize the inflow artifact from the blood, and achieve better longitudinal coverage. No spatial saturation was applied. Total acquisition time was 6 min 23 s, to obtain a mask image and 30 view-shared phases with a temporal resolution of 10.6 s. Coincident with the third phase, a bolus of 0.1 mmol/kg Gd- DTPA (Gadovist, Bayer Schering, Berlin, Germany) was administered using a power injector at the rate of 3 mL/s followed by a 20 mL saline flush. The CE-MRA data was obtained by subtracting the mask image from the multi-phase acquisition. In addition, for plaque component determination, the following sequences were also performed in the coronal plane: pre- and post-contrast 3D T_1_-weighted (T_1_w) delays alternating with nutation for tailored excitation (DANTE)-prepared fast spin echo [24], 3D time of flight (TOF) and 3D direct thrombus imaging (MR-DTI, inversion time of 300 ms) [25]. Detailed imaging parameters are listed in Table 1. Total scanning time was approximately 30 min.

**Table 1:** Summary of imaging parameters for the multi-contrast MRI protocol at 3T

Sequence	3D TOF	Pre/post-contrast BB T_1_w 3D FSE	3D DTI	4D DCE/MRA
Flip angle (°)	20	Variable flip angle	30	20
TE/TR (ms)	2.2/5.9	16.9/540	4.2/8.6	1.5/3.9
FOV (mm × mm × mm)	140 × 140 × 64	140 × 140 × 67	160 × 160 × 66	140 × 140 × 62
Acquired pixel size (mm × mm × mm)	0.5 × 0.5 × 2.0	0.6 × 0.6 × 1.4	1.0 × 1.0 × 1.0	0.6 × 0.6 × 1.4
Reconstruction pixel size (mm × mm × mm)	0.5 × 0.5 × 2.0	0.3 × 0.3 × 0.7	0.3 × 0.3 × 1.0	0.3 × 0.3 × 0.7
NEX	2	2	1	1
Receiver bandwidth (±kHz)	31.25	62.5	25	62.5
Acquisition time	1 min 35 s	2 × 6 min 26 s	4 min 42 s	6 min 23s^a^

*TOF* time of flight, *BB* black blood using delays alternating with nutation for tailored excitation (DANTE) preparation, *FSE* fast spin echo, *DTI* direct thrombus imaging, *DCE* dynamic contrast-enhanced, *MRA* MR angiography, *TE* echo time, *TR* repetition time, *FOV* field of view, *NEX* number of excitations

^a^Temporal resolution 10.6 s

### Image analysis

The T_1_w and DCE images were reformatted into the axial plane with a 0.7 mm slice thickness using a dedicated workstation (AW4.6, GE Healthcare, Buc, France). For each patient, the carotid arteries on both sides were analyzed. Carotid vessel wall and lumen boundaries at each slice were manually drawn according to the T_1_w images, using a DICOM viewer (OsiriX 5.5.2, Pixmeo, Geneva, Switzerland). Plaque was defined as a focal wall thickness ≥1.5 mm [26]. The subtracted CE-MRA image at the fifth phase, which had the highest image contrast in the carotid artery, was used for morphological analysis. Plaque surface morphology was classified as either ulcerated/irregular or smooth using the maximum intensity projection (MIP) of CE-MRA images, based on previous published standards [10], by two experienced reviewers, both having more than 2 years of experience in carotid imaging. The two reviewers were blinded to the patients clinical information and made their judgement independently. The classification result from the first reviewer was used for further analysis. Luminal stenosis was measured for each plaque on both CE-MRA source image and 3D pre-contrast T_1_w images separately according to the North American Symptomatic Carotid Endarterectomy Trial (NASCET) criteria [23, 27]. The stenosis measured from CE-MRA source image was used for further analysis. This method has a reported high intra- and inter-observer reproducibility [8]. IPH was defined as bright pixels on the DTI image.

### Pharmacokinetic modeling

Images from the 4D data were processed using the University of Washington (Seattle) vasa vasorum imaging (VVI) tool [28]. This approach firstly applies a Kalman Filtering Registration and Smoothing (KFRS) algorithm, to reduce the noise level in the image and correct patient motion [29]. A two-compartment Patlak model is then used to generate a parametric map known as the “vasa vasorum image (VVI)” showing partial plasma volume (*v*_p_) in shades of red and transfer constant (*K* ^trans^) in shades of green. The relationship between blood and tissue signal concentration is modeled as follows:

1 $$C_{\text{t}} \left( t \right) = v_{\text{p}} C_{\text{p}} \left( t \right) + K^{\text{trans}} \int\limits_{0}^{t} {C_{\text{p}} \left( {t^{\prime } } \right){\text{d}}t^{\prime } }$$

where $C_{\text{t}}$ and $C_{\text{p}}$ represent the contrast agent concentration in the tissue and blood plasma, respectively. Lumen and wall boundaries manually segmented from T_1_w images were copied to the VVI. Manual adjustments were performed, as necessary to co-register the ROIs to the VVIs, by the reviewers who were blinded to the final results. Further details of the analysis can be found in Kerwin et al. [28]. Adventitial PK measurements were calculated by averaging all the pixels along the wall boundary. Plaque PK measurements were calculated by averaging all the pixels between the wall and lumen boundary. The overall PK parameters of each plaque were calculated as the mean value across all the plaque containing slices. To test the interrater reproducibility of this method, two reviewers preformed the analysis independently for all of the patients.

The PK parameter differences were evaluated using linear and non-linear assumptions of the signal intensity and Gd concentration. The following equations were used to calculate the Gd concentration:

2 $$I = M_{0} \frac{{\sin \left( \alpha \right)\left( {1 - e^{{ - \frac{\text{TR}}{{T_{1} }}}} } \right)}}{{1 - \cos \left( \alpha \right)e^{{ - \frac{TR}{{T_{1} }}}} }}e^{{ - \frac{\text{TE}}{{T_{2}^{*} }}}}$$

3 $$r\left[ {\text{Gd}} \right] = \frac{1}{{T_{1} }} - \frac{1}{{T_{1,0} }}$$

where Eq. (2) calculates the pixel *T*_1_ value according to its signal intensity based on the spoiled gradient echo sequence [30]. $\alpha$ is the flip angle and *M*_0_ is a constant value. Equation (3) calculates the Gd concentration (mmol/L) according to the pixel *T*_1_ changes. *T*_1_ and *T*_1,0_ (s) are the current and native pixel *T*_1_ values (blood *T*_1,0_ = 1500 ms and vessel wall *T*_1,0_ = 1000 ms from the literature value [31] were used). The corrected Gd concentration was then applied again to the 28 arteries using Eq. (1). The calculated PK parameters were then compared with the results from the linear assumption.

### Statistical analysis

The statistical analysis was performed using R (version 3.2.2). The Shapiro–Wilk’s test was used to test normality assumptions. The Kappa statistic was performed to evaluate interrater agreement in ulceration detection. The Student’s *t* test was used to compare the PK parameters for smooth versus ulcerated/irregular plaques defined by the first reviewer, and also to compare hemorrhage and non-hemorrhage plaques. Pearson’s correlation was used to assess the relationship between PK parameters and luminal stenosis. The concordance correlation coefficient (CCC) was reported to assess the relationship between the stenosis measured by CE-MRA and 3D T_1_w images. The CCC and Bland–Altman plots were used to compare the differences between linear and non-linear assumptions of image signal and Gd concentration. The inter-rater agreement in PK parameters was assessed using the intraclass correlation coefficient (ICC). A Chi-squared tests was used to compare proportions of patients with IPH. Parametric distributions were presented as mean ± SD. *p* values <0.05 were considered statistically significant.

## Results

Of the 21 patients, three patients did not finish the examination due to discomfort, four carotid arteries were excluded from data analysis due to motion artefacts and four arteries were excluded due to occlusion. The rest of the images were of diagnostic quality, and used for further analysis. Atherosclerotic plaques were identified in the remaining 28 arteries, with a mean luminal stenosis of 44% (range 11–82%). Fourteen plaques were classified as ulcerated/irregular and fourteen were classified as smooth. In the 28 arteries analyzed, two independent reviewers agreed in 26 (93%) cases for plaque surface morphology classification ($\kappa = 0.79$; 95% CI 0.56–1.00). The PK parameters (*K* ^trans^ and *v*_p_ in adventitia and plaque) were normally distributed (*p* > 0.05). For the luminal stenosis, the correlation between CE-MRA and 3D T_1_w was good (*r* = 0.89).

**Fig. 1:**
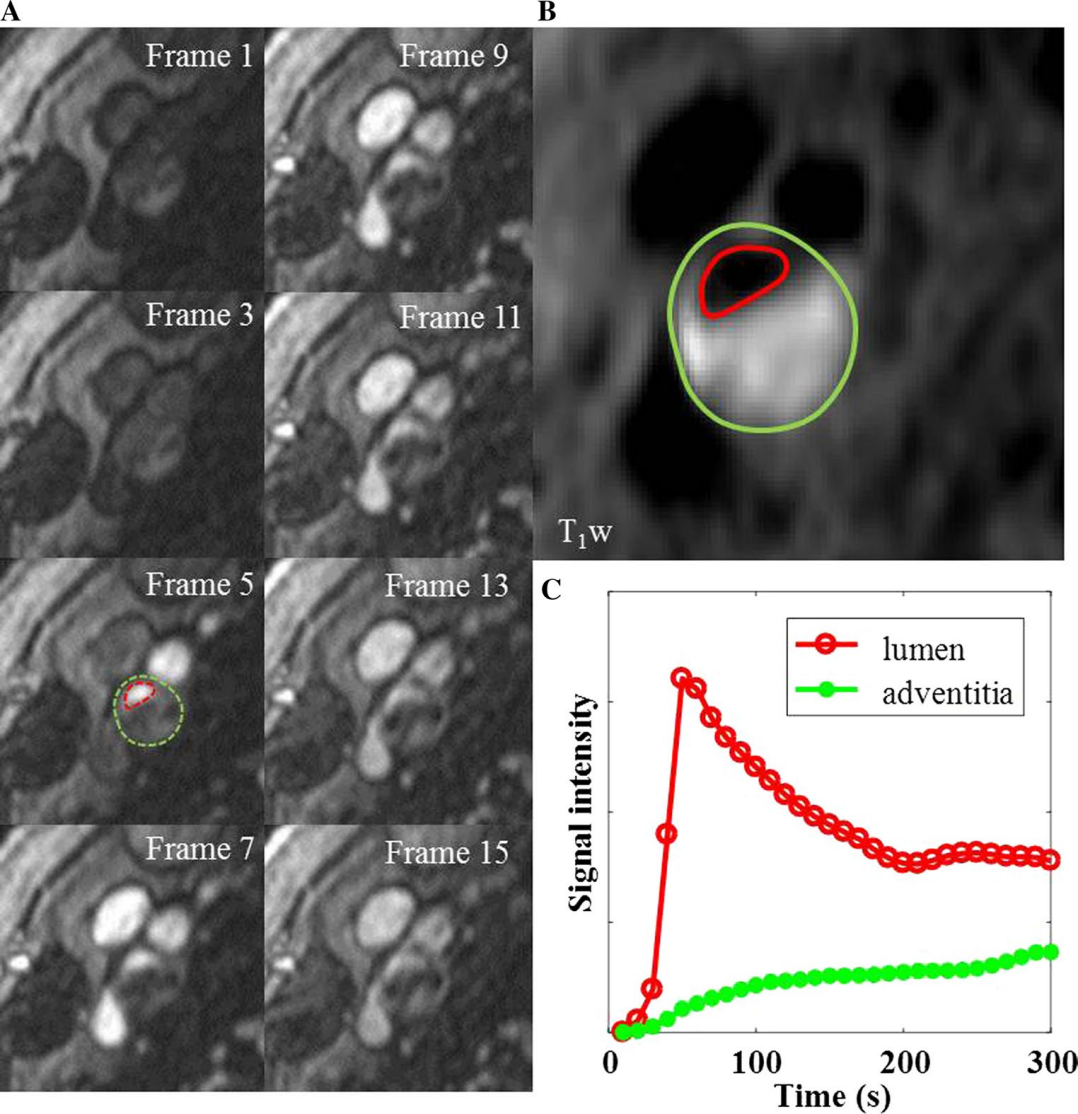
**a** Shows DCE images at different time frames at a single slice location within an ICA branch of the plaque. The *dashed red and green lines* in *frame 5* show the boundary of carotid lumen and adventitia. **b** Shows the corresponding *black* blood T_1_w image with *red and green lines* delineating the lumen and wall boundary. **c** Represents the mean signal intensity time course within the lumen (*red*) and adventitia (*green*)

**Fig. 2:**
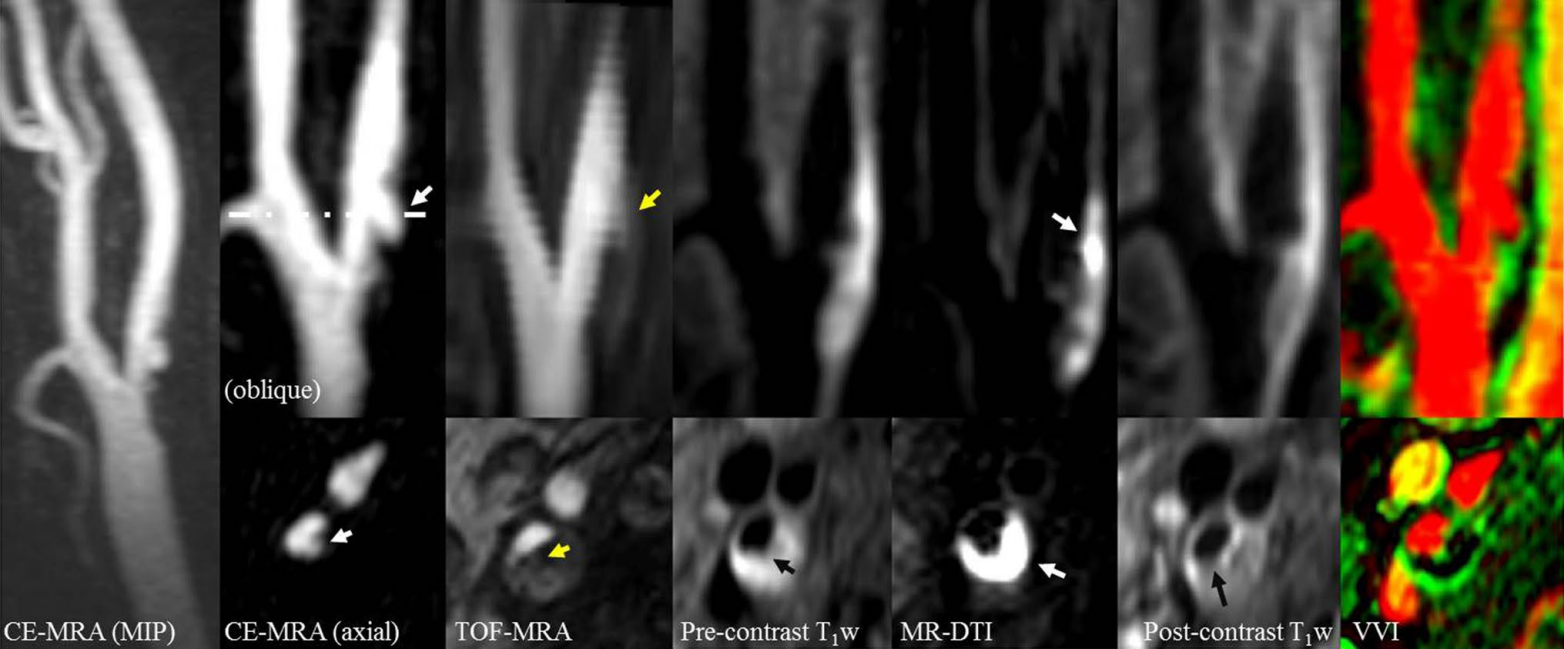
An ulcerated plaque in the multi-contrast MR protocol, including MIP from CE-MRA, oblique and axial reformat of CE-MRA, TOF-MRA, T_1_w, DTI, CE-T_1_w and VVI. The *white arrow* in CE-MRA suggests the ulcer arises perpendicularly from the lumen. The ulcer can be clearly seen on the CE-MRA images, while it is not clearly visible on the TOF-MRA images. The pre-contrast T_1_w image shows the lumen surface irregularity, and a thin or ruptured FC can be seen on the post-contrast T_1_w images (*black arrow*). The hyperintense area on MR-DTI represents a large intraplaque haemorrhage/thrombus (*white arrow*). The VVI has the range of *v*_p_ from 0 to 65% and *K* ^trans^ from 0 to 0.5 min^−1^ which shows a high *K* ^trans^ region at the adventitia

The images in Fig. 1 are from a symptomatic patient, with a 52% stenosis on the right side. The plaque contains a large intraplaque hemorrhage (IPH). Figure 1c shows the intra-luminal and adventitial signal change with time following contrast agent administration. Figure 2 is from the same artery and shows the multi-contrast MR images and calculated VVI. An ulceration can be clearly seen on the CE-MRA images, but is not so apparent on the 3D TOF-MRA. Surface irregularity can be observed in both pre- and post-contrast T_1_w images. The MR-DTI shows a large region of IPH. The VVI shows regions of high *K* ^trans^ around the ulceration and at the adventitia boundary, with a relatively low *K* ^trans^ inside the IPH, which is in agreement with a previous study [32].

**Fig. 3:**
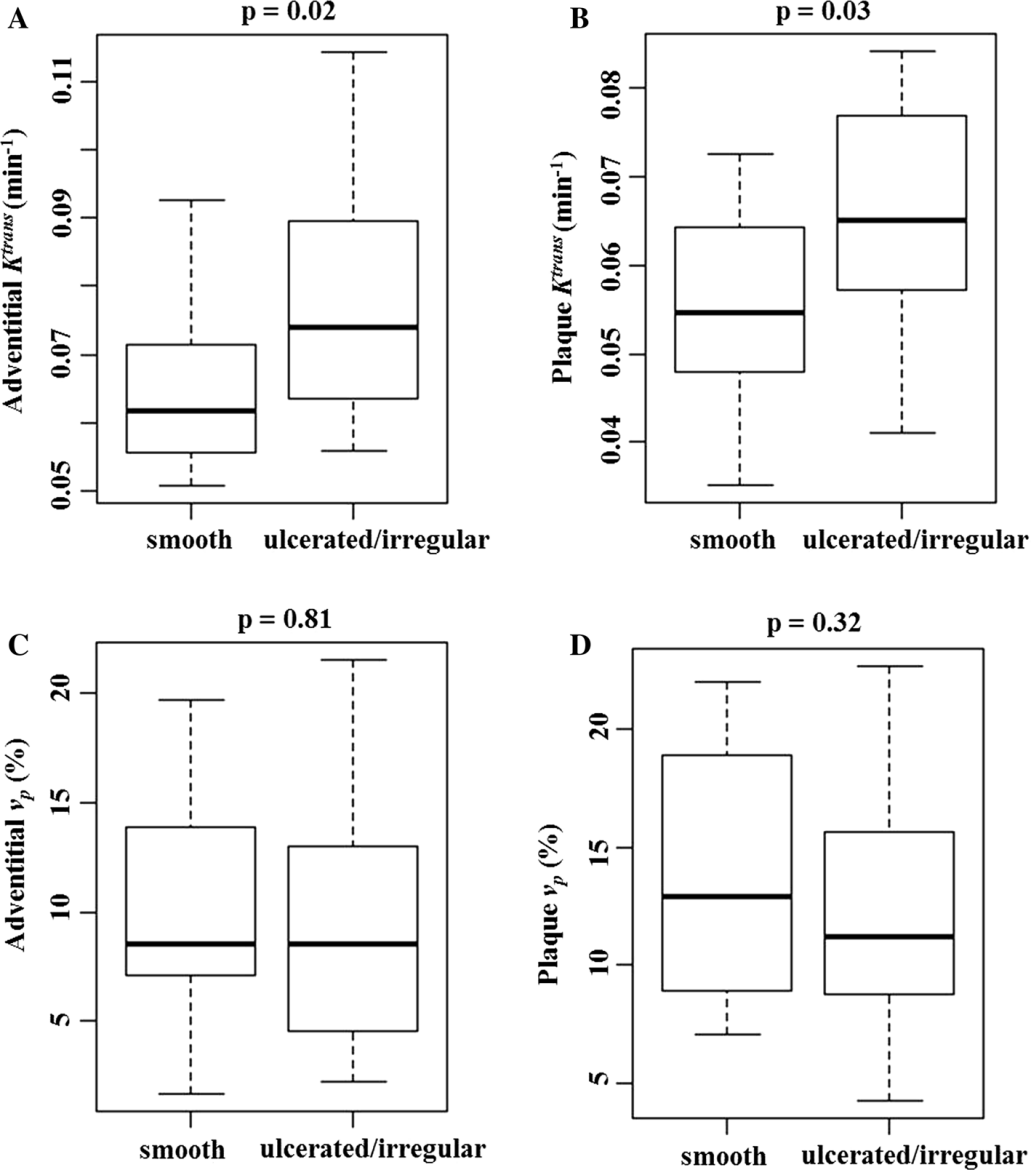
Comparison of pharmacokinetic parameters in smooth and ulcerated/irregular plaque

The PK values from the linear assumption were used for the statistical analysis. Adventitial and plaque *K* ^trans^ in ulcerated/irregular plaques were significantly higher compared to smooth plaques (Fig. 3a, b, adventitial *K* ^trans^ 0.079 ± 0.018 vs. 0.064 ± 0.011 min^−1^, *p* = 0.02; plaque *K* ^trans^ 0.065 ± 0.013 vs. 0.055 ± 0.010 min^−1^, *p* = 0.03, respectively). Whilst *v*_p_ demonstrated no significant difference between groups (Fig. 3c, d, adventitial *v*_p_ 9.3 ± 5.3% vs. 9.8 ± 4.8%, *p* = 0.81; plaque *v*_p_ 13.5 ± 5.5% vs. 11.8 ± 4.9%, *p* = 0.32, respectively).

**Fig. 4:**
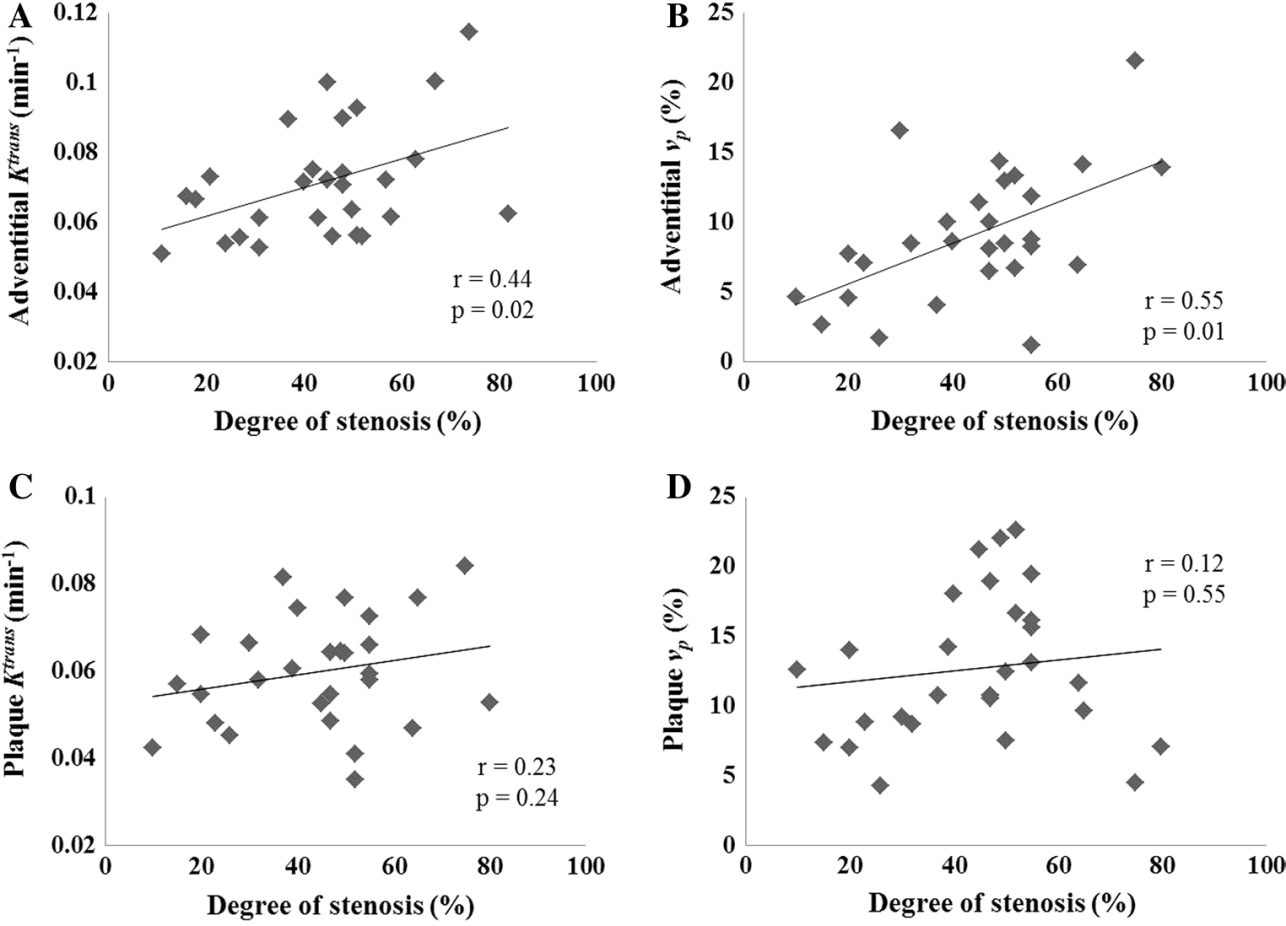
**a** Shows the significant correlation between stenosis and adventitial *K* ^trans^. **b** Shows the significant correlation between stenosis and adventitial *v*_p_. There is no significant correlation between plaque *K* ^trans^ or *v*_p_ and stenosis (**c**, **d**)

Positive correlations between adventitial *K* ^trans^ and *v*_p_ with the degree of stenosis were observed (Fig. 4a, b, *r* = 0.44, *p* = 0.02 for adventitial *K* ^trans^; *r* = 0.55, *p* = 0.01 for adventitial *v*_p_, respectively). While no significant correlation was observed for plaque *K* ^trans^ or *v*_p_ with stenosis (Fig. 4c, d, *r* = 0.23, *p* = 0.24 for plaque *K* ^trans^; *r* = 0.12, *p* = 0.55 for plaque *v*_p_, respectively).

**Fig. 5:**
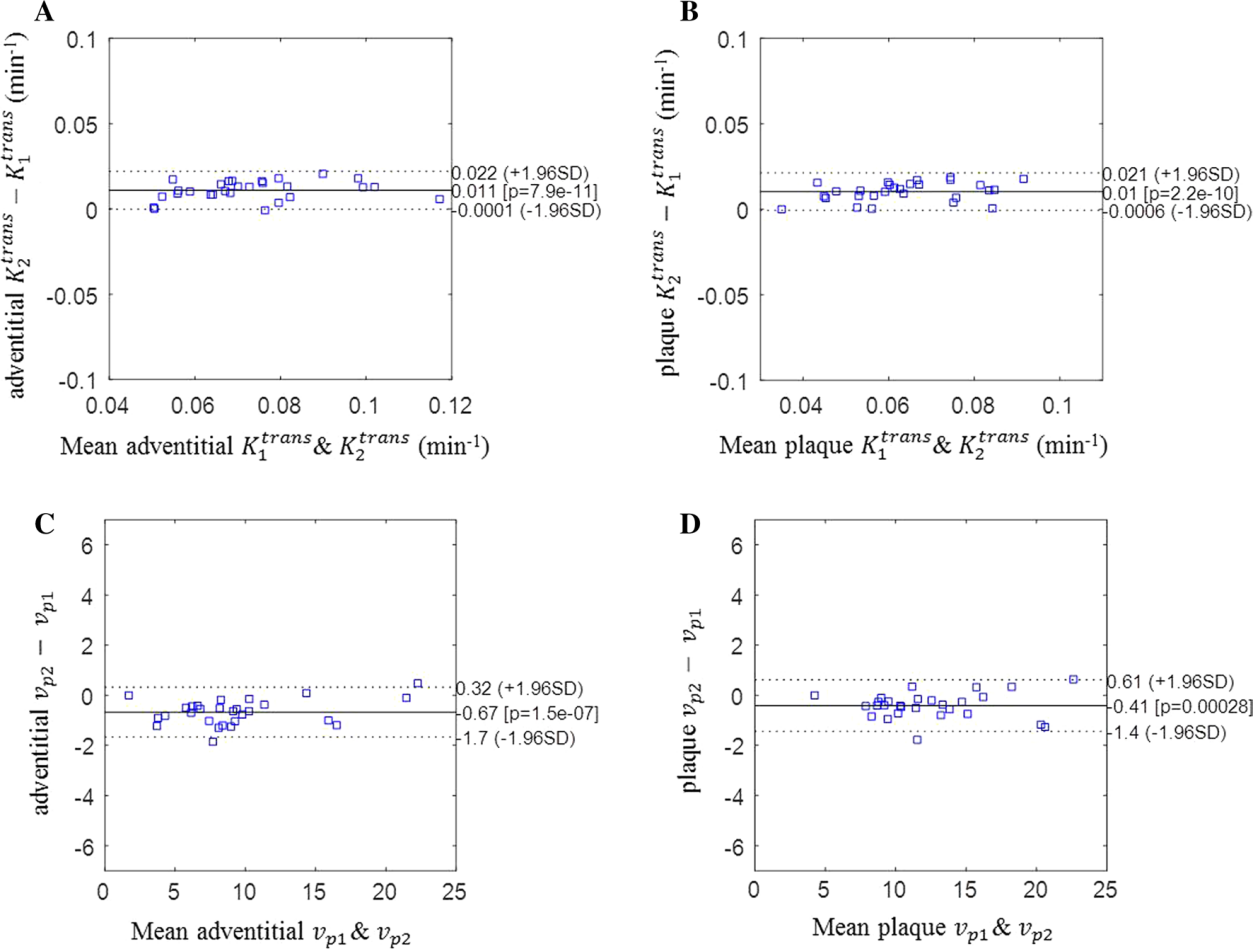
Bland-Altman plots of the PK parameters derived after modelling the linear and non-linear relationships of signal intensity and Gd concentration. There is a significantly bias in PK values when using linear and non-linear assumption of image signal to Gd concentration. Note that $K_{1}^{\text{trans}}$ and *v*_p1_ represent the linear model, and $K_{2}^{\text{trans}}$ and *v*_p2_ represent the non-linear model

The CCC comparing the linear and non-linear assumptions for adventitial *K* ^trans^, plaque *K* ^trans^, adventitial *v*_p_ and plaque *v*_p_ were: 0.95, 0.93, 0.99 and 0.99, respectively. There was highly significant concordance between the linear or non-linear assumptions (all *p* < 0.001), though there is a significant bias in PK values when using the linear and non-linear assumption of image signal to Gd concentration. Figure 5 demonstrated the Bland–Altman plots comparing both assumptions.

IPH was found in eight plaques. Within this group, six arteries had ulcerated plaques and two had smooth plaques. In the 20 plaques without IPH, we noted that smooth plaques were more common (12 had smooth plaques), however, these respective proportions were not significantly different (*p* = 0.28). Plaques with hemorrhage have higher mean adventitial *K* ^trans^ values compared with plaques with no hemorrhage (0.078 ± 0.019 vs. 0.069 ± 0.015 min^−1^), however the difference here was not significant (*p* = 0.2).

The agreement for the four measured DCE parameters between the two reviewers was good. The ICC for adventitial *K* ^trans^, plaque *K* ^trans^, adventitial *v*_p_ and plaque *v*_p_ are 0.88, 0.93, 0.81 and 0.71, respectively.

## Discussion

To the best of our knowledge, this is the first application of a 4D sequence to acquire both high spatial and temporal resolution DCE-MRI and CE-MRA of the carotid artery, allowing for combined morphological and functional assessment of carotid atherosclerosis from a single contrast administration. The results show that adventitial and plaque *K* ^trans^ were higher in ulcerated/irregular plaques compared to smooth plaques. Adventitial *K* ^trans^ and *v*_p_ were also positively correlated with the degree of luminal stenosis.

Carotid plaque ulceration, characterized as an intimal defect with the intraplaque component exposed to the lumen [9], can form emboli and thrombosis leading to ischemic neurologic symptoms. Sitzer et al. showed plaque ulceration and lumen thrombus are the main sources of cerebral microemboli [9]. Multiple studies have also shown that plaque ulceration is associated with cerebral infarcts, TIA and amaurosis fugax [8, 11]. In this study, plaque enhancement could be observed in both smooth and ulcerated plaques and the derived VVI showed significant differences in PK parameters between both of these plaque types. The results demonstrate that ulcerated plaques are more prevalent to neovascularization and possibly inflammation. Plaque ulceration or fibrous cap discontinuity may induce more inflammatory cell infiltration into the plaque, which in turn further weakens the fibrous cap and eventually causes the plaque to rupture. This suggests quantitative analysis of DCE-MRI could provide more information than conventional contrast-enhanced morphological images in identifying lesions at higher risk.

While only adventitial *K* ^trans^ and *v*_p_ are correlated with luminal stenosis, no significant correlation was observed between whole plaque PK values and the stenosis. This might be due to a lower sensitivity as a result of averaging PK parameters across the whole plaque region. While various plaque components such as IPH, lipid core and fibrous tissue have different PK values [32], simple averaging of PK parameters across the plaque region may be insufficient for quantitative analysis and risk stratification. A positive correlation between luminal stenosis and adventitial *K* ^trans^ and *v*_p_ was observed in this study (Fig. 4a, b), indicating a relationship between plaque growth and adventitial neovascularization. These microvessels within the atherosclerotic lesion have shown close association with inflammatory cells [33] which indicate that these could serve as a pathway for the macrophage entry into the plaque that leads to chronic inflammation hence promoting plaque development.

The presence of intraplaque hemorrhage (IPH) has also been found to be associated with increased adventitial *K* ^trans^ [34]. In our study, plaques with IPH had higher adventitial *K* ^trans^, however the difference was not significance. The reason for this may due to lack of statistical power, as only a small number of plaques (eight) contained IPH. Although the exact etiology of the IPH is still unclear it is suggested that most likely the rupture of fragile and leaky neovessels that vascularize the developed plaque leads to IPH [35].The histopathological evidence also supports the relationship of IPH to the presence of neovessels [36]. In our study, IPH was absent in most of the plaques. The correlation between PK parameters and surface morphology also applies to the plaques without IPH, indicating ulceration and a high degree of stenosis were two other factors, apart from IPH, which could present with increased plaque neovascularization and inflammation.

The use of multi-contrast MRI allows for detailed analysis of plaque composition and vulnerability assessment, which could provide more insight than simple luminal stenosis measurements [37]. This study demonstrates that a combined 4D DCE-MRI/MRA acquisition in combination with multi-contrast 3D sequences can aid in the identification of high-risk plaque features. Our study also demonstrates that these features can be obtained in a clinically acceptable scan time of approximately 30 min.

There are several limitations in this study. Firstly, no histological validation was performed on the plaques. However the correlation between DCE-MRI and neovascularization as well as inflammation has been demonstrated in previous studies [19, 21, 28], and CE-MRA is considered reliable for observing plaque surface morphology [13, 16]. The second limitation is that only a small patient cohort was included in this study. As discussed above, the *K* ^trans^ difference between IPH groups was not significant, this might be due to the resultant lack of statistical power. Thirdly, the view-ordering used in this study could smooth the temporal information, especially during the injection period. However, this is not considered to be a significant effect and other studies have used similar analysis for various DCE applications, including breast [38, 39], prostate [40] and also animal models [41]. The last limitation is that a thick coronal imaging slice (1.4 mm) was used in this study. This might bring errors in calculating the PK values for the thin vessel wall. Future protocol design could consider increasing axial resolution for a more precise analysis.

## Conclusion

This study investigated the relationship between PK modeling with carotid plaque surface morphology by simultaneously acquiring 4D DCE-MRI and CE-MRA. Adventitial and plaque *K* ^trans^ from DCE-MRI analysis is significantly higher in ulcerated plaques than smooth ones. Also, adventitial *K* ^trans^ and *v*_p_ are correlated with luminal stenosis.
